# Multidrug resistance characterization in multicellular tumour spheroids from two human lung cancer cell lines

**DOI:** 10.1186/s12935-015-0200-6

**Published:** 2015-04-24

**Authors:** Raúl Barrera-Rodríguez, Jorge Morales Fuentes

**Affiliations:** Departamento Bioquímica y Medicina Ambiental., Instituto Nacional de Enfermedades Respiratorias, Clza. Tlalpan, 4502, Col Sec. XVI., Mexico, 14080 Mexico; Jefe del Serv. Neumología. Hospital Central Del Sur, Pemex, Mexico, Mexico

**Keywords:** Multicellular tumour cancer spheroids, Multicellular resistance, Multidrug resistance genes, Drug cytotoxicity, Three-dimensional culture, Lung cancer

## Abstract

**Background:**

Most of the knowledge about the mechanisms of multidrug resistance in lung cancer has been achieved through the use of cell lines isolated from tumours cultivated either in suspensions of isolated cells or in monolayers and following exposition to different cytostatic agents. However, tumour cell lines growing as multicellular tumour spheroids (MTS) frequently develop multicellular resistance in a drug-independent form. The aim of this study was to characterize the phenotypic and functional differences between two human NSCLC cell lines (INER-37 and INER-51) grown as traditional monolayer cultures versus as MTS.

**Methods:**

After 72 hours treatment with anticancer drugs, chemosensitivity in monolayers and tumour spheroids cultures was assessed using MTT assay. Reverse transcription-polymerase chain reaction was employed to detect the mRNAs of multidrug resistance-related genes. The expression of P-gp was analyzed by immunohistochemical staining and cell cycle profiles were analyzed using FACS.

**Results:**

The results indicate that when grown as MTS each lung cancer cell line had different morphologies as well as and abrogation of cell proliferation with decrease of the G_2_/M phase. Also, MTS acquired multicellular resistance to several chemotherapeutic agents in only a few days of culture which were accomplished by significant changes in the expression of MDR-related genes.

**Conclusion:**

Overall, the MTS culture changed the cellular response to drugs nevertheless each of the cell lines studied seems to implement different mechanisms to acquire multicellular resistance.

## Introduction

Lung cancer is the most common cause of cancer-related deaths in the world, causing more than one million deceases worldwide each year [1]. Despite of the major advances in patient management, chemotherapy and radiotherapy made over the past decades, long-term survival is only obtained in 20-35% of the patients with advanced non-small cell lung cancer [2,3], and even with the use of new target therapies, the response has not been as spectacular as expected [4].

The major obstacle in lung cancer treatment is the intrinsic resistance that these tumours present to chemotherapy [5]. As a result, much of what we know about the mechanisms of multidrug resistance in lung cancer has been obtained through the use of cell lines isolated from tumours cultivated either in suspensions of isolated cells or in monolayers (2D) and exposed to different cytostatic agents [6]. However, monolayer cell cultures lack the three-dimensional cell arrangement that may participate in the emergency of the drug-resistant phenotype. To overcome these limitations, multicellular tumour spheroids (MTS) have been developed to reproduce *in vitro* the histological and architectural organization of the tumour tissue [7]. Interestingly, when tumour cells are cultured as MTS, they spontaneously develop resistance to several chemotherapeutic drugs, a phenomenon known as "multicellular resistance" (MCR) [8,9]. Therefore, MTS appear to be more resistant to drugs than monolayer cultures [10] indicating that the multicellular tissue architecture and the altered cell-cell contact may play a role in the mechanism of MCR acquisition [11].

Several hypotheses have been proposed to explain the acquisition of MCR. One is the development of non-proliferating quiescent cells in central areas of the tumour, characterized by extreme microenvironment conditions and hypoxia [12,13]. A correlation between the appearance of the quiescent cell subpopulation in large MTS and P-glycoprotein (P-gp) mediated resistance has been suggested [14]. However, the contribution of P-gp and the other MDR-related gene products has not been extensively analyzed yet.

Albeit, a great number of studies suggest that only one cellular mechanism confers MCR, a few of them try to explain the real contribution of different pathways in the MTS cultures; even less studies are focused in lung cancer tumours. Therefore, we decided to grow human lung cancer cell lines as MTS to analyze the changes in drug sensibility as well as the molecular mechanisms that develop to acquire MCR. Our results showed that the MTS culture of two lung cancer cell lines induced MCR to several chemotherapeutic drugs after only a few days of culture. The MCR in lung cancer cell tumours was accomplished by the generation of quiescent cells and strong changes in the expression of MDR-related genes. However to acquisition of MCR by each multicellular tumour spheroid seems to depend on the specific nature of each the cell line.

## Results

As monolayers, both lung cancer cells showed different growth patterns, with INER-37 cells growing as adherent cells forming a proper monolayer and a doubling time of 79 hours, whereas INER-51 cells showed a loosely adherent phenotype and a doubling time of 31 hours.

### Histological appearance

**Figure 1:**
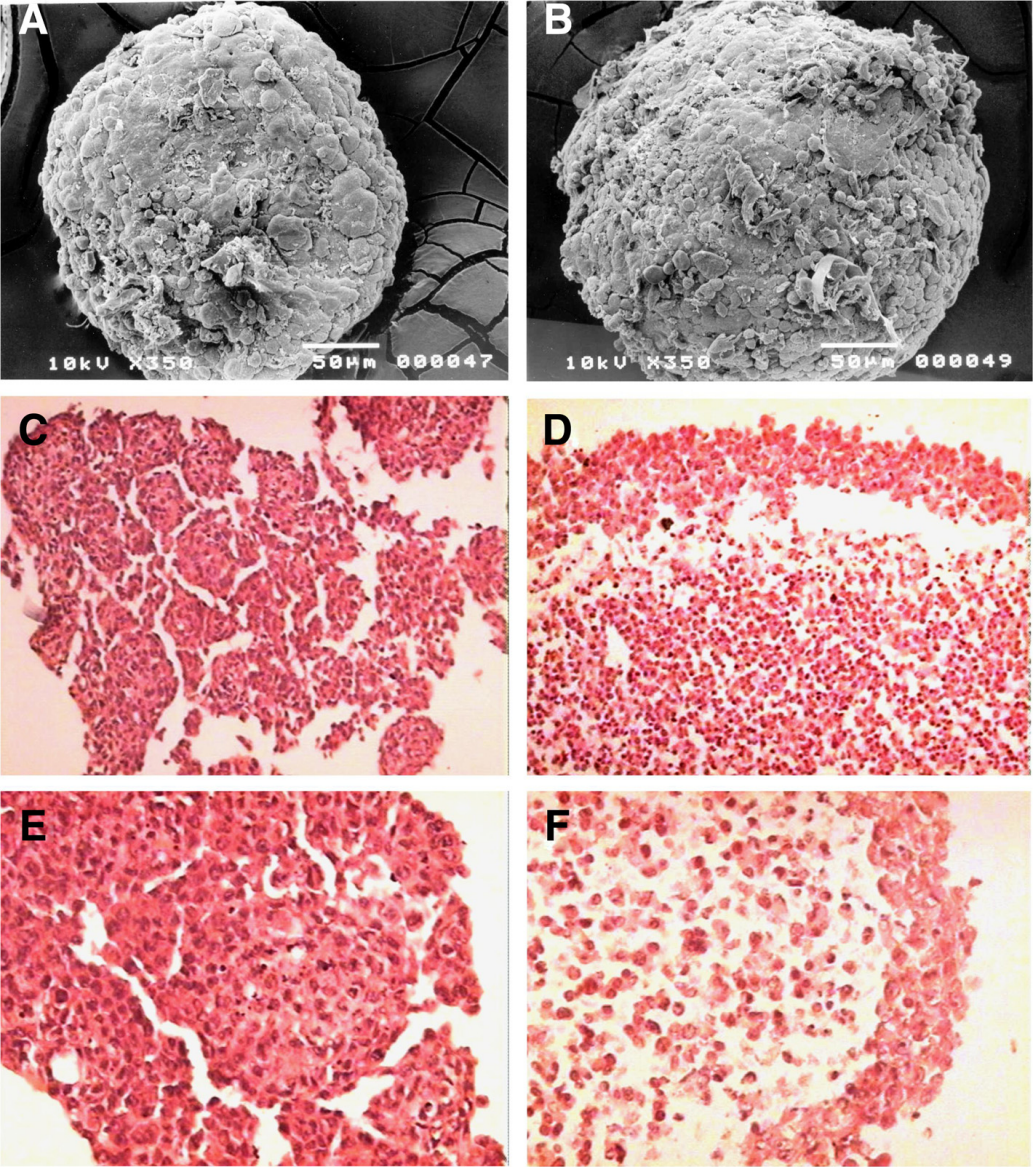
INER-37 and INER-51 MTS morphology. Left panels correspond to INER-37 spheroids while right panels are INER-51 spheroids. Panels** A** and **B** show an ultramicrographic photograph of the respective MTS after 72 hours of *in vitro* culture (200X). Panels **C **to **F** are different H&E staining microphotographs of the MTS. For panels **C ** and **D**, the amplification was 400X, and for **E** and **F** it was 1200X.

When cultured in a non-adhesive environment, both lung cancer cells can aggregate and differentiate into MTS. Ultramicroscopic examination of MTS showed cell aggregates with great compactness (Figure 1A and B). However, by hematoxilin-eosin staining, both lung cancers MTS were formed by completely different structures. Thus, INER-37 spheroids were formed by small aggregates with cells tightly attached to each other, while reaching an approximately diameter of 550 ± 25 μm (Figure 1C and E), whereas INER-51 spheroids were formed by larger aggregates, which acquired a maximal diameter of 1,314 ± 30 μm (Figure 1D and F). In INER-51 MTS, two zones were clearly visible: the peripheral zone composed by a rim of tightly attached cells and the inner zone where cells were more loosely attached forming a lax tissue and showing multiple empty spaces. During 72 hrs of spherule culture, evidence of necrotic zones were not seen in either of the two MTS.

### Monolayers and MTS proliferation

**Figure 2:**
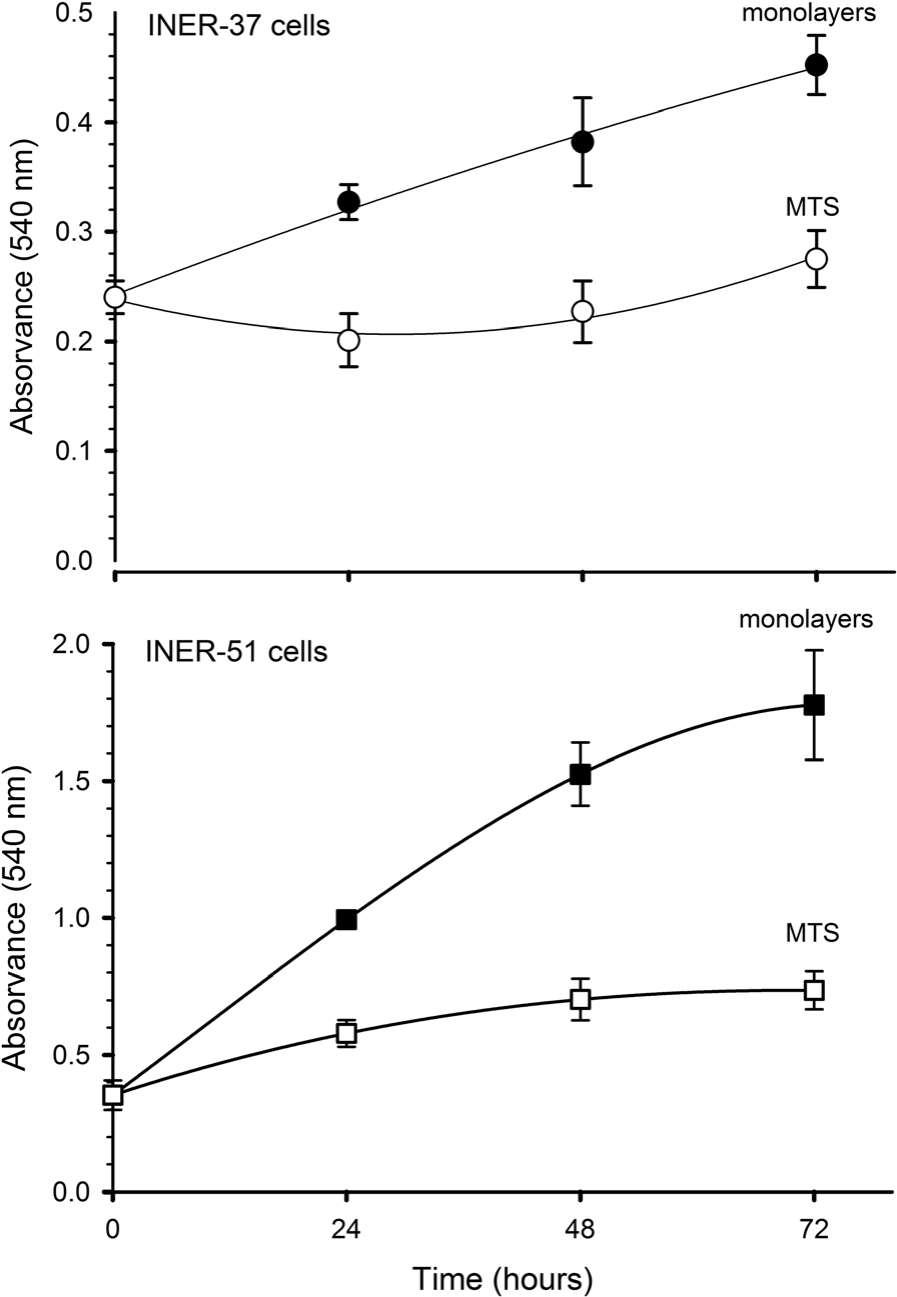
Changes of growth patterns of human lung cancer cell lines grown as monolayers (black symbols) or multicellular tumour spheroids (white symbols). Increase in cell proliferation was determinate by MTT assay every day. Data are mean ± standard deviation (SD) of three independent experiments.

Several reports have mentioned that tumour cells growing as MTS have a significant impact in their proliferation rate. Thus, it was important to find out if under our culture conditions, the cell proliferation was also altered. The results showed a dramatic decrease in cell proliferation when cells were cultured as MTS when compared with cell lines grown as monolayer (Figure 2). This decrease was evident as early as 24 hrs of culture and was kept during the whole experimental period.

### Cell cycle analyses

**Figure 3:**
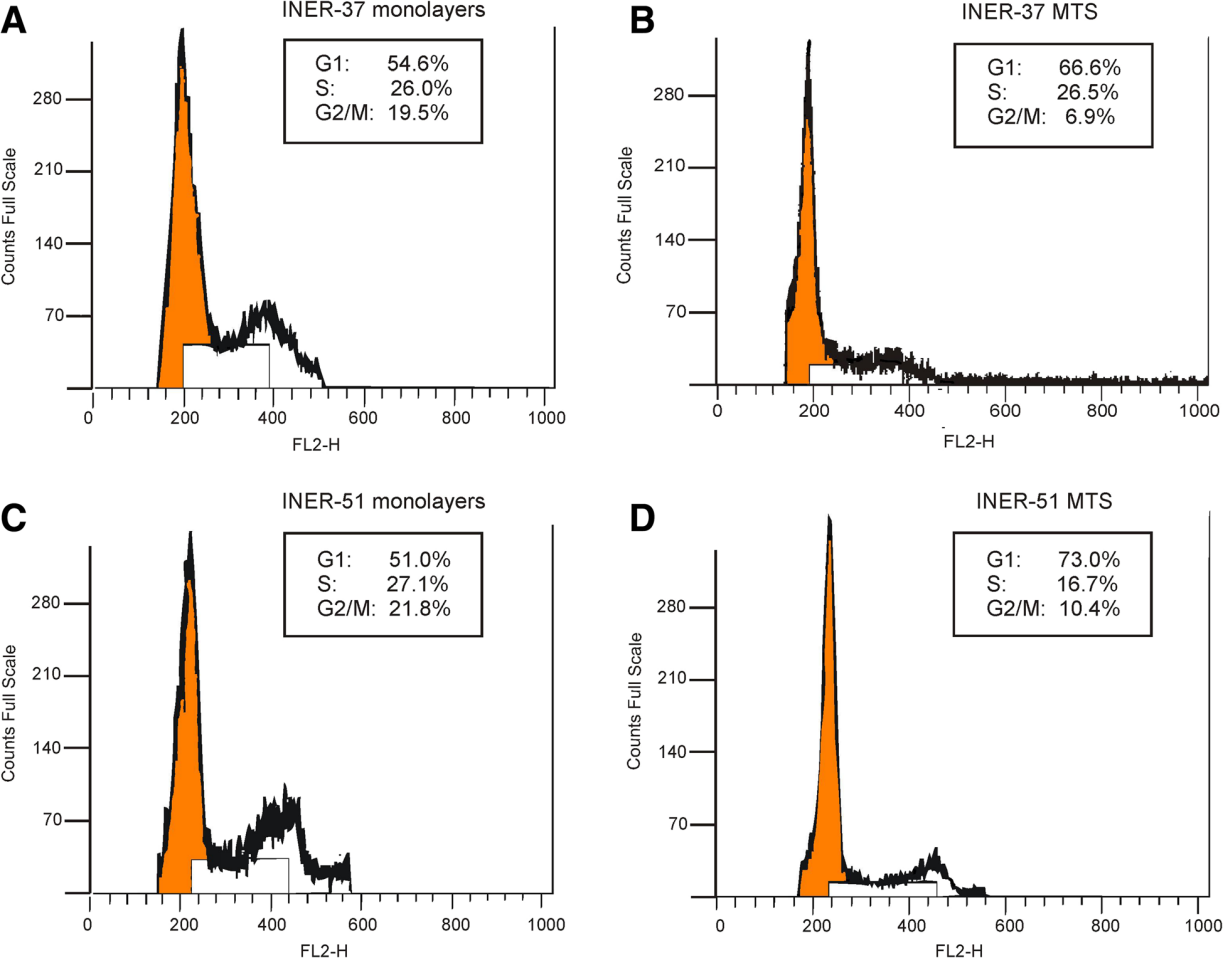
Cell cycle analysis of lung cancer cell lines grown as monolayer or MTS. Cell lines INER-37 or INER-51 as monolayer **(A, C)** or MTS **(B, D)** respectively, were disassociated with 0.25% trypsin, washed, and stained with propidium iodide for DNA content analysis as described in Material and methods. Flow cytometry was then used to generate cell cycle profiles. Each flow cytometry plot depict the mean percentage of G1 (2*n*), S and G2/M (4*n*) fraction population. Each value represents the mean of at least two experiments.

To determine whether the ending of cell growth in the spheroid culture correspond to the cells arrest in a particular phase of the cell cycle, we analyzed a fraction of DNA by flow cytometry. Figure 3 shows cytometric plots of DNA for monolayers and MTS, showing cell cycle distribution of the analysed cells. Interestingly, a consistently higher proportion of cells were in G_1_ phase and a significantly reduced amount of cells was at the G_2_/M phase throughout the growth of the MTS as compared to monolayer exponential phase. Thus, the population on G_2_/M decreased in the MTS condition for both cell lines: 19.5% (monolayer) *vs* 6.9% (spheroid) for INER-37 and 21.8% (monolayer) *vs* 10.4% (spheroid) for INER-51, with a consequent change in the percentage of the cell cycle phases.

### Drug resistance profiles of monolayes and MTS

**Figure 4:**
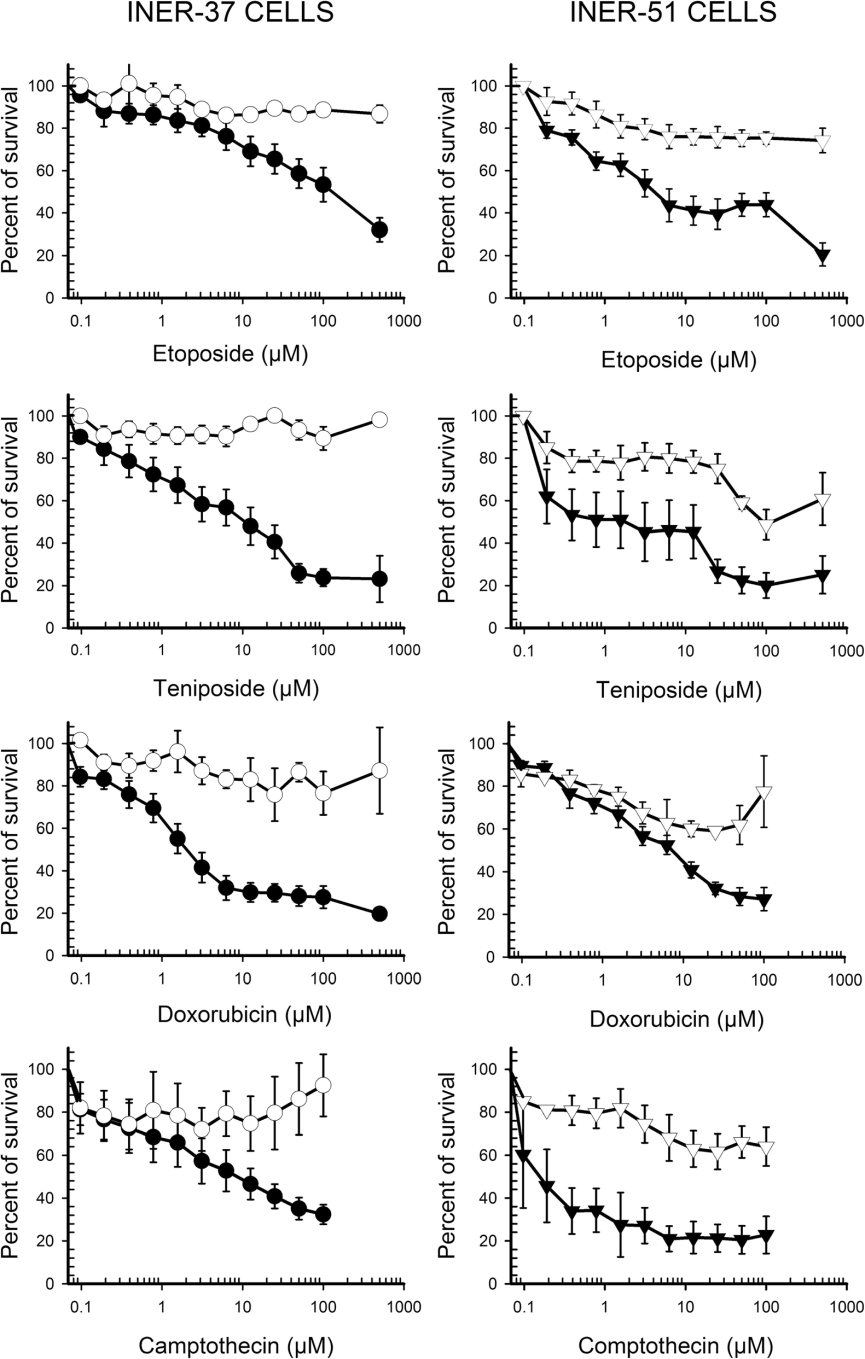
Dose–response curves to cytotoxic agents of lung cancer cell lines grown as monolayer or spheroids. Cell lines INER-37 or INER-51 as monolayer (●) or MTS (○) were exposed to varying concentrations of etoposide, teniposide, doxorubicin or camptothecin, for 72 h. Drug cytotoxicity was determined by incubation with the tetrazolium dye MTT and measurement of absorbance at 540 nm. Each point value is the average of six independent experiments.

In order to know how the culture condition affected drug response, both monolayers and MTS were exposed to a panel of several chemotherapeutic drugs. In almost all cases, MTS were significantly less sensitive than monolayers to all drug concentrations tested. For example, when INER-37 and INER-51 MTS were exposed to 500 μM of etoposide, the survival rate was higher than 90% and 73%, whereas in monolayers the survival rate was 10% and 20% respectively (Figure 4). A similar pattern was seen when MTS were exposed to teniposide, camptothecin and doxorubicin with the survival rate always higher than in monolayers. Interestingly, no significant change the activity of cisplatin was found between MTS vs monolayer in two lung cancer cell lines.

A detailed contrast of IC_50_ values of MTS to several cytotoxic agents is show in the Table 1. In all cases, the increase in drug resistance was evident with IC_50_ values being higher in MTS than in monolayers. On INER-51 MTS the relative resistance respect to monolayers was higher than INER-37 values because INER-51 cells were intrinsically more sensitive to anticancer agents.

**Table 1:** **IC**_**50**_ **mean values for different drugs tested in monolayer and MTS of lung cancer cell lines**

**Drug**	**INER-37 2D (μM).**	**INER-37 MTS (μM).**	***t-*** **student**	**INER-51 2D (μM).**	**INER-51 MTS (μM).**	***t*** **-student**
Etoposide	17.9	>500	0.0001	9.9	>500	<0.0001
Teniposide	9.5	>500	0.0003	3.9	>500	<0.0001
Doxorubicin	3.9	>100	<0.0001	7.9	87.3	0.014
Camptothecin	9.1	>100	0.004	0.01	>100	<0.0001
5-Fluorouracil	300	>500	NS	5.0	>500	<0.0001
Methotrexate	300	>500	NS	2.6	>500	<0.0001
Cytosine-Arabinoside	300	>500	NS	0.95	>500	<0.0001
Cisplatin	29.0	74.8	0,033	74.8	25.5	0,003

Five thousand cells per well were plated in 96 well plate previously precoated with or without agarose and the drug cytotoxicity was determinate by MTT assay. Each IC_50_ value is the average of six independent experiments. The symbol (>) indicates that the IC_50_ was not reached at the highest concentration tested. NS: Not significant.

Drug resistance can be related to cell-interactions because cell lines arrested by serum deprivation showed only a discrete increase in resistance to etoposide with IC_50_ values of 45 μM and 26 μM in INER-37 and INER-51 respectively, in contrast to higher resistance in multicellular spheroids (*data not shown*).

### Expression of MDR-1 and multidrug resistance associated genes

**Figure 5:**
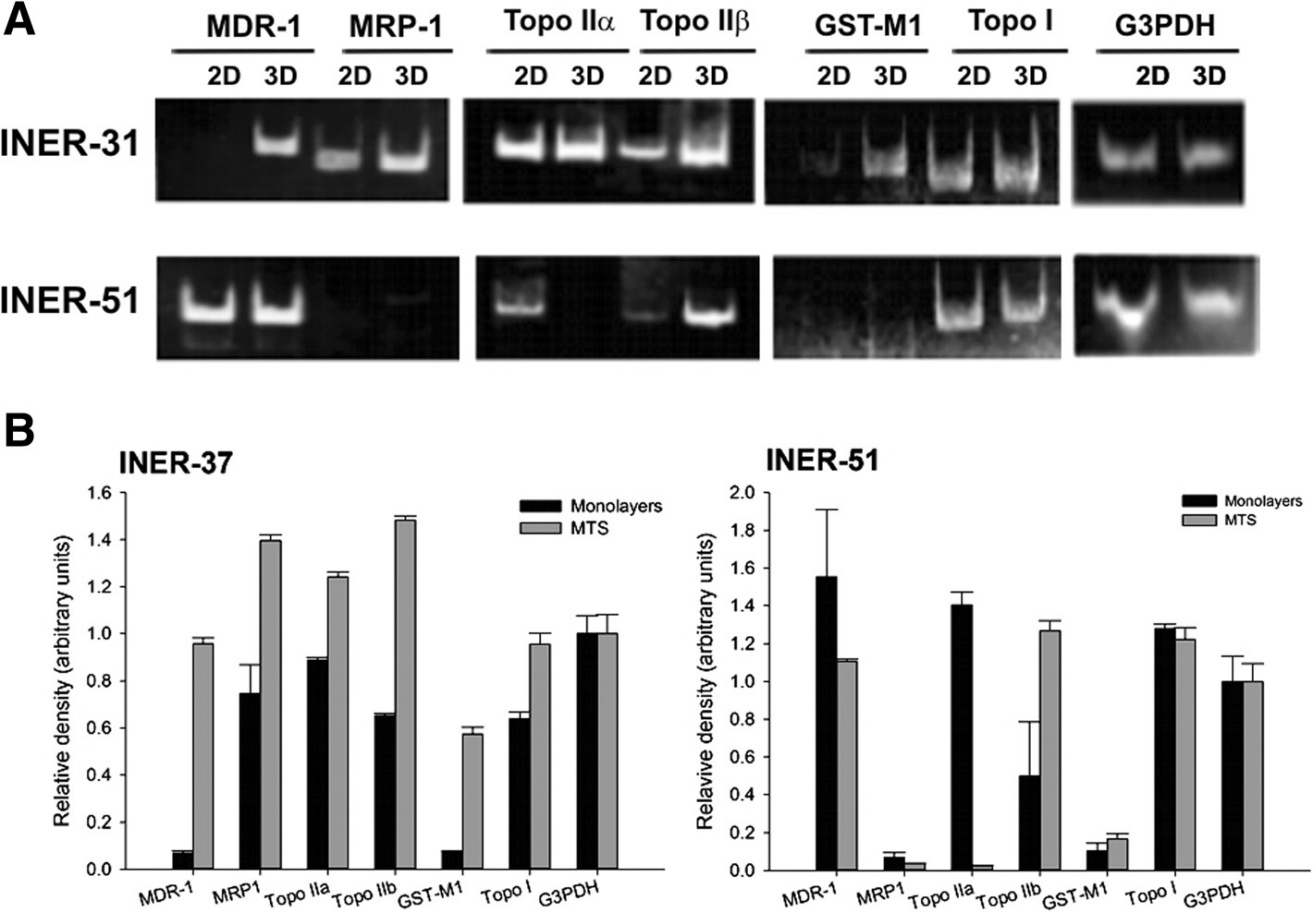
Reverse transcriptase-polymerase chain reaction (RT-PCR) analysis of mRNA expression for MDR-related gene products on lung cancer cell lines grown as monolayers or MTS. The expression of the following genes was evaluated: *MDR-1, MRP-1, Topo IIα, Topo IIβ, GST-μ, Topo I,* and *G3PDH*. Amplification products were electrophoretically separated in polyacrylamide gels and revealed by etidium bromure staining. The analysis of G3PDH expression was used as constitutive control for the integrity of RNA molecules. **A)** Representative RT-PCR and **B)** Densitometric analysis of RT-PCR band intensities for MDR-related genes normalized by G3PDH. Data are mean ± standard deviation (SD) of three independent experiments.

With the aim of determining if MTS formation affected the expression of genes associated with the acquisition of resistance, we analysed several of them. RT-PCR analysis shows different patterns of mRNA expression for each MTS. Thus, INER-37 monolayers did not express *MDR-1* gene but it was tuned-on when cells were cultured as MTS (Figure 5). For INER-51 cells, the expression of *MDR-1* was equal and independent of the culture conditions. Contrarily, *MRP-1* expression was up-regulated on INER-37 spheroids but in INER-51 cells, neither monolayers nor MTS expressed it. For *Topo II*α gene, INER-37 MTS did not show changes in its expression but this gene was down-regulated in INER-51 MTS. Furthermore, expression of *Topo II*α on INER-51 monolayer was less than in INER-37 cells. For *Topo II*β gene, both MTS cell lines up-regulated its expression. Other genes associated with drug resistance were also analyzed. Thus, the *GST-*μ expression in INER-37 was positive and independent from the culture, but INER-51 cells did not express this gene under any culture condition. In the case of *Topo I*, a similar expression was observed for the two cell lines, which was independent of the culture conditions. The expression of *G3PDH* gene was used to verify the integrity of transcripts in all the experiments.

### Immunostaining of P-gp in INER-37 MTS

**Figure 6:**
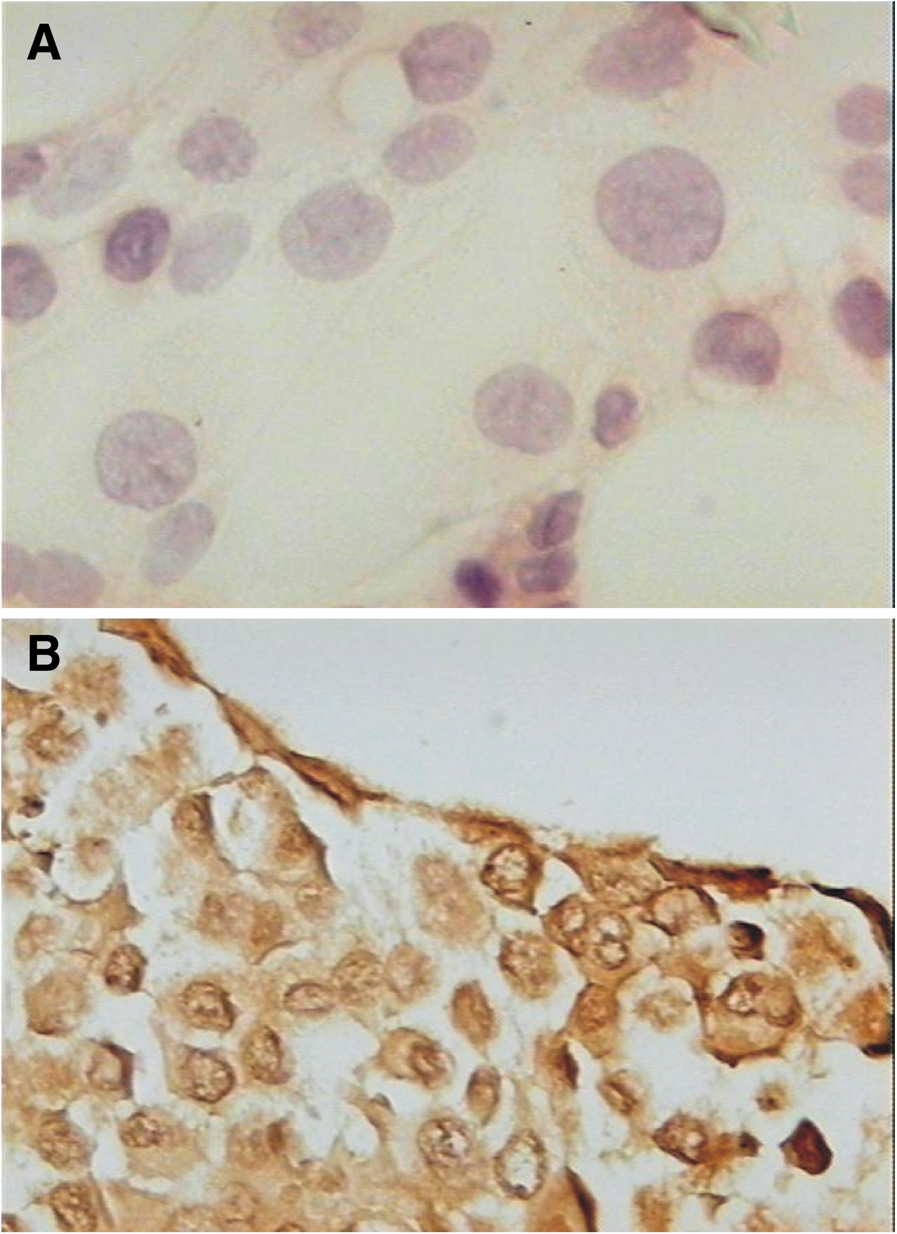
Immunocytochemistry of INER-37 cells. **A)** Monolayer cells or **B)** multicellular tumour spheroids of INER-37 were labelled with anti-P-gp (C494 antibody, DAKO) and immunostained with anti-rabbit IgG linked to biotin-HRP, followed by DBA to develop colour. Photomicrographs were taken with a Nikon microscope at 620X of total magnification.

To verify if MDR-1 transcript on INER-37 MTS correlated with a P-gp expression, we performed an anti-P-gp immunohistochemistry using C494 antibodies (which recognized one intracellular epitope). Figure 6 shows representative whole-mount MTS labels with anti-P-gp antibody. Positivity to P-gp was evident in all cells forming the INER-37 MTS. For INER-51, no differences in immunochemical label to P-gp were observed between monolayers and MTS (*data not shown*).

## Discussion

By using a low adhesion model to produce multicellular tumour spheroids, we were able to generate reproducible-sized spheroids of two lung cancer cell lines. Each MTS showed its own characteristics in growth and shape, with spheroids reaching up to 500 μm in diameter.

Several studies show that the acquisition of drug resistance in MTS is mainly due to low penetration, a phenomenon that becomes more evident in spheroids up to 400 μm [15,16]. The INER-37 MTS were formed by tightly attached cells, resembling a classical model described for others spheroids, where cellular aggregation as well as the presence of tortuous 3-D scaffold structures may limit the drug exposure to some cells and could be in part responsible for chemoresistance [17]. In contrast, in INER-51 MTS, the peripheral zone was formed by a narrow multicellular layer rim with multiple intercellular spaces, allowing the free diffusion of drugs toward the centre of the spherule. Thus, a limited drug diffusion associated with large spherule size is difficult to consider both MTS models because of the tissue arrangement observed in INER-51 spheroids. Differences in the penetration rate for anticancer agents as a function of cellular adhesion and packing density were shown by Grantab *et al.* in a model of multicellular layers using two epithelioid and round subclones of HCT-8 colon carcinoma cell line [18]. In addition, the morphological observation and the evidence that ^14^C-doxorubicin penetration through multicellular layers show a free drug transit without any multicellular interference [19,20], as well as spheroid permeability data obtained for various drugs including doxorubicin [12], suggest to us that impaired penetration of anticancer agents through MTS is not a general phenomenon to explain MCR, as has been mentioned before.

The spherule formation also resulted in different depth-dependent micro-environmental conditions, such as gradient of oxygen and pH [21], nutrients [22], and accumulation of catabolic products in the centre of the spheroid [23], which produce differential cell nutrition based on cell´s localization and the appearance of quiescent cells. Under these conditions, smaller spheroids consist exclusively of proliferating cellular-cycling cells, while larger spheroids contain extended areas of quiescent cells arrested in the G_0_/G_1_ phase of the cell cycle. It is commonly known that quiescent cells are relatively resistant toward anti-cancer drugs and this has been explained mainly by a reduced susceptibility of DNA damage [24] or increasing capability of DNA repair [25]. In our case, both INER-37 and INER-51 MTS showed a decrease in the cell proliferation, with a proportional rise of cell population in G_0_/G_1_ phase that could be responsible for MCR. Interestingly, host cell microenvironment or cell-cell interactions seem to be greatly important in MCR generation, as both lung cell lines arrested by serum deprivation showed only a discrete increase in resistance to etoposide (*data not shown*).

Furthermore, much evidence shows that multidrug resistance is associated with the expression of multidrug related genes [26,27], especially in the up-regulated expression of MDR-1 gene [28]. Gene expression of specific sets of genes has been shown to be modulated by cell culture architecture [29], possibly in relation to signals effectively transduced upon interaction of adhesion molecules with their counter receptors [30]. In our case, lung cancer MTS showed significant changes in transcript production of several of these MDR related genes. Thus, INER-37 MTS turned on MDR-1 gene and its product, P-gp, helped us understand the increase in drug resistance. Therefore, drug resistance of INER-37 MTS could be matched with a more classic mechanism, where the quiescent state is closely related to the expression of P-gp, constituting the first line barrier to obstruct drug from diffusing the centre of the spherule. Increased P-gp expression and MDR phenotype in the absence of any previous exposure to chemotherapy drugs have been found in monolayers cultured under conditions that mimic the micro-environment present in quiescent cells layers of MTS i.e. confluence [31], hypoxia [32], glucose deprivation [33] and increased hydrostatic pressure and acidic pH [34]. Conversely, in INER-51 MTS neither up-regulated *MDR-1* nor expression of *MRP-1/GST-*μ transcripts was observed. In a previous work, we found that INER-51 MTS showed a more efficient P-gp activity, which was responsible of maintaining lower retention levels of doxorubicin. However, P-gp expression contributes to keeps lower levels of doxorubicin in the MTS; the mechanisms that govern the MCR seems to be different because the lack of P-gp-expression only showed a minor impact of resistance to several chemotherapy drugs, suggesting that other non-P-gp mechanisms are also operating [35]. In addition, constitutive expression of BCRP1 has been identified in INER-51 cells; however this mechanism *per se* is not enough to confer drug resistance phenotype to the broad spectra of chemotherapeutic agents studied.

The degree of tumour responsiveness to different chemotherapeutic drugs has also been associated to the relative level of expression of Topo IIα and Topo IIβ isoforms. Reduction of transcription of topo II to one third of the initial activity, occurs within 1 day of growth as spheroids, and declines by a similar proportion again over 3–4 days [36]. Our results show that INER-37 MTS did not modify the transcription levels of Topo IIα, as it was expressed to the same extent in both culture types. However, in previous studies we reported that INER-37 cells have a mutation in the Topo IIα gene, which encodes for a cytoplasmic enzyme [37]. In agreement with our results, Oloumi *et al.* found that etoposide resistance correlated with changes in subcellular distribution of Topo IIα in outer cells of V79 spheroids and xenograft tumors [38]. Thus, in INER-37 MTS, the multicellular resistance seems to be the result of a combination of mechanisms: through expression of P-gp pump and by an altered intracellular distribution of the Topo IIα enzyme. In INER-51 MTS, the results were different, since Topo IIα seems to be down regulated. For these cells, the low expression of the Topo IIα gene and the activity of P-gp could explain the resistance to Topo II inhibitors (like doxorubicin, etoposide and teniposide).

Unlike Topo IIα, most evidence has shown that the transcription of Topo IIβ is not cell cycle regulated [39]. However, the transcript of Topo IIβ was up-regulated in the two types of MTS, suggesting some dependency on the cell cycle. Our results are similar to those of Drake *et al.*, who found an increase in the levels of Topo IIβ and a decrease in Topo IIα, both associated with a state of quiescence [40].

Several other mechanisms of drug resistance seem to be intimately related to the three dimensional cell growth. One of them is the interaction between MRP-1 and GST enzymes [41]. The GST enzymes can chemically alter several types of drugs that will serve as substrates for MRP-1 that contribute to extrude them out of the cell [42]. In INER-37 MTS, the up-regulated expression of *MRP-1* working with *GST-*μ could be important in drug resistance to doxorubicin. Interestingly, in INER-51 MTS these were not the mechanisms employed because non of these genes was expressed. However, as we do not know much about the possible interaction of these two systems and considering the fact that we only studied one member of the GST family, it is reasonable to believe that other members of the GST family might interact with other members of the MRP family members to induce MCR [43]. Takagi *et al.* found that the gene expression profile in the MTS was different from that in the monolayers; however, it was similar to that in the solid tumor tissue [44].

## Conclusion

The culture of lung cancer lines as MTS results in the acquisition of MCR mechanisms which confer adaptive advantages of the multicellular growth given by microenvironmental conditions. Many of them are inter-related or independent of each other, but may exist simultaneously in cancer cells or subpopulation of cells, producing an overall drug-resistant phenotype. Consequently, each cell line seems to implement different mechanisms to acquire MCR. Further research in defining the mechanisms that regulate drug-resistant phenotype *in vitro* may provide key information for the development of novel treatment strategies to overcome drug resistance.

## Material and methods

### Chemicals

Etoposide (VP-16), teniposide (VM-26), 5-Fluorouracil (5-FU), doxorubicin hydrochloride (DXR), cytosine β-D-arabinofuranoside (Ara-C), methotrexate hydrate (MTX), cyclophosphamide monohydrate), Cisplatin and (*S*)-(+)-Camptothecin (CPT), 3-[4,5-Dimethyl-thizol-2-yl]-2,5-diphenyltetrazolium bromide (MTT) were pursed from Sigma-Aldrich Co., St. Louis MO. Drugs were dissolved in dimethyl sulfoxide to 100 mM stock solutions that were stored at 4°C. These were diluted in water to 1 mM working stocks prior to the assays.

### Lung cancer cell lines

In this study we used two cancer cell lines designated as INER-37 and INER-51. Both tumour cell lines were established in our laboratory from plural effusions of patients with primary lung cancer without previous chemotherapy treatment. Pleural effusion samples were collected after obtaining written and signed informed consent, in accordance with the Helsinki Declaration.

By cytological studies, INER-37 tumour cells were identified as a well-differentiated adenocarcinoma while INER-51 tumour cells were characterized as a poorly differentiated adenocarcinoma.

The EGFR-mutation status in exons 18, 19, 20 and 21 of the EGFR gene was negative for both cell lines.

### Culture of monolayers and MTS

The lung cancer cell lines INER-51 and INER-37 were grown as monolayer cultures in RPMI-1640 medium at 37°C in 5% CO_2_. The medium was supplemented with 10% fetal calf serum (Thermo Scientific*HyClone), 2 mM L-glutamine, 100 units/ml of penicillin and 100 μg/ml of streptomycin. On reaching 75% confluency, the tumour cells were washed with phosphate-buffered saline (PBS) and then removed from the flask by the addition of 0.25% trypsin (Invitrogen™, Life Technologies) and washed twice with PBS. To obtain MTS, approximately 5–7 × 10^3^ cells/well were seeded onto 1% agarose-coated 24-well/plate with RPMI-1640 complete medium. Three-dimensional cell cultures were routinely grown for 72 hours to acquire MTS larger than 500 μm of diameter. For cell proliferation, both monolayers and MTS growths were determined by MTT essay.

### MTS cytology and immunocytochemistry

Tumour spheroids were examined cytologically via routine paraffin slicing techniques after fixation on absolute ethanol. The thickness of the hematoxylin-eosin stained serially sliced paraffin section was of 5 μm. For immunological analysis, monolayer of cells (grown on microscope slides) and multicellular tissue samples were reacted with antibody anti-P-gp (C494 antibody, Dako Inc.) at a dilution of 1:50.

Detection of primary antibody was done with a detection system kit (*VECTASTAIN® ABC systems*) with a secondary goat anti-mouse immunoglobulin-G linked to biotin-HRP, followed by DBA to develop colour.

### Scanning electron microscopy (SEM)

Multicellular spheroids were washed with PBS and fixed in 2.5% glutaraldehyde for 2 h. The spherules were then post-fixed on the plate with 1% OsO_4_ in 0.1 M cacodylate buffer pH 7.3 before dehydrating with ethanol, critical point drying, mounting on carbon stubs, and coating with gold before viewing under a JEOL 7401 series FEGSEM (Jeol Ltd).

### Drug resistance in monolayers or MTS

The level of resistance to drugs was determined with the use of the MTT assay as previously described [35]. For monolayers, 7 × 10^3^ cells/well were plated in 96-well/plate (Costar, USA) and drugs were added at different concentrations per well. In the case of the MTS, they were obtained as described above and were fed with fresh complete medium containing different drug concentrations. After 72 hours, the culture medium was removed and MTT reagent diluted in PBS was added to obtain a final concentration of 2 mg/ml. After incubation for 4 hours, individual spheroids surrounded with formazan crystals were transferred into 1.5 ml eppendorf tubes. Cells in monolayers were washed carefully with PBS once. Both monolayers and MTS crystals were dissolved by addition of 100% DMSO for 20 min with occasional shaking. Absorbance at 540 nm was measured using an automated microplate reader (Labsystem Multiskan MS, Finland). In each experiment, the drug determination was analyzed in six individual wells. Cell survival was estimated as a percentage of the corresponding control. Drug-cytotoxicity was assayed by the IC_50_, corresponding to the 50% decrease in cell survival rate compared to the no-drug treated cultures.

### In vitro reverse transcriptase-PCR assay

Total RNA was extracted from the cell lines with Trizol reagent (Invitrogen™, USA) according to the manufacturer's instructions. Single stranded cDNA was synthesized by reverse transcription from 5 μg of total RNA using Superscript™ RNAse Reverse Transcriptase (Invitrogen™, USA) and oligo-dT_16–18_. The amplification was performed in a final volume of 25 μl, containing 0.5 μl cDNA, 50 pM of each oligonucleotide primer, 30 μM of each dNTPs, 2.5 units of Taq DNA polymerase, 1.5 mM MgCl_2_, 20 mM Tris–HCl (pH 8.4) and 50 mM KCl. Amplification was carried out in a Thermal Cycler (Programmed Thermal Controller, model PTC-100, MJ Research Inc., USA) for 35 cycles of denaturalisation at 94°C for 1 min, annealing at 55–60°C for 2 min, and polymerising at 72°C for 3 min. The PCR primers and expected product size were as follows: For MDR-1, forward: 5'-cccatcattgcaatagcagg-3' and reverse: 5'-gttcaaacttctgctcctga-3 [150 bp]; MRP1, forward: 5'-tctctcccgacatgaccgagg-3' and reverse: 5'-ccaggaatatgatgccccgacttc-3' [140 bp]; topoisomerase IIα, forward: 5'-tttaaggcccaagtccagttaaac-3' and reverse: 5'-gtataacaatatcatcaagattgt [343 bp]; topoisomerase IIβ, forward: 5'-gaagtgttcactagtaaaatacagt-3' and reverse: 5'-cataatctttccatagcgtaaggtt-3' [336 bp]; topoisomerase I, forward: 5'-aagcagaggaagtagctacg-3' and reverse: 5'-gctcatctgtttccgagctt-3' [206 bp]; GST-μ, forward: 5'-gaactccctgaaaagctaaag-3' and reverse: 5'-gttgggctcaaatatacggtgg-3' [250 bp]; G3PDH, forward: 5'-tggggaaggtgaaggtcgga-3' and reverse: 5'-gaaggggtcattgatggcaa-3' [110 bp].

### Cell cycle analysis

Tumour cells were fixed in ethanol 80% at −20°C. Samples were rehydrated in two steps to 100% water. Tumour cells were digested by pepsin (5 mg/ml) in 0.9% saline solution, pH 1.5, at 37°C for 20 min. Samples were washed with PBS and 1 × 10^6^ cell nuclei were incubated with trypsin (30 μg/ml) for 10 min. Immediately, soybean trypsin inhibitor (277 μg/ml) and ribonuclease (55 μg/ml) were added and incubated for additional 10 min at room temperature. Finally, propidium iodide (150 μg/ml) was added to the nuclear preparation and incubated in darkness at 4°C for 10 min. Nuclei were analysed in a FacSort flow cytometer with argon laser adjusted to emit light at 488 nm. Histograms of the area under the curve of FL2 (FL2 H) were obtained. Data analysis was performed with ModFit software using RFIT mathematical model (Becton-Dickenson). Calf thymus and chicken erythrocyte nuclei were used as external standards in order to validate DNA measurements in diploid cells.

### Statistical analysis

Statistical analyses were performed using Student’s *t* test. The differences were considered significant for *p* values of <0.05.

## Abbreviations

P-gp: P-glycoprotein
MCR: Multicellular resistance
MDR: Multidrug resistance
MTS: Multicellular tumour spheroids
PBS: Phosphate-buffered saline
RT-PCR: Reverse transcriptase-polymerase chain reaction
bp: Base pair(s)

## References

[1] Ferlay J, Soerjomataram I, Ervik M, Dikshit R, Eser S, Mathers C et al. GLOBOCAN 2012 v1.0, Cancer Incidence and Mortality Worldwide: IARC CancerBase No. 11. Lyon, France: International Agency for Research on Cancer; 2013. http://globocan.iarc.fr.

[2] Schiller JH, Harrington D, Belani CP, Langer C, Sandler A, Krook J (2002). Eastern cooperative oncology group. Comparison of four chemotherapy regimens for advanced non-small-cell lung cancer. N Engl J Med.

[3] Johnson DH, Schiller JH (2014). Recent clinical advances in lung cancer management. J Clin Oncol.

[4] Lee JK, Hahn S, Kim DW, Suh KJ, Keam B, Kim TM (2014). Epidermal growth factor receptor tyrosine kinase inhibitors vs conventional chemotherapy in non-small cell lung cancer harboring wild-type epidermal growth factor receptor: a meta-analysis. JAMA.

[5] Arriagada R, Auperin A, Burdett S, Higgins JP, Johnson DH, NSCLC Meta-analyses Collaborative Group (2010). Adjuvant chemotherapy, with or without postoperative radiotherapy, in operable non-small-cell lung cancer: two meta-analyses of individual patient data. Lancet.

[6] Breen L, Keenan J, Clynes M (2011). Generation of lung cancer cell line variants by drug selection or cloning. Methods Mol Biol.

[7] Sutherland RM (1988). Cell and environment interactions in tumor microregions: the multicell spheroid model. Science.

[8] Olive PL, Durand RE (1994). Drug and radiation resistance in spheroids: cell contact and kinetics. Cancer Metastasis Rev.

[9] Desoize B, Jardillier J (2000). Multicellular resistance: a paradigm for clinical resistance?. Crit Rev Oncol Hematol.

[10] Hirschhaeuser F, Menne H, Dittfeld C, West J, Mueller-Klieser W, Kunz-Schughart LA (2010). Multicellular tumor spheroids: an underestimated tool is catching up again. J Biotechnol.

[11] Ekert JE, Johnson K, Strake B, Pardinas J, Jarantow S, Perkinson R (2014). Three-dimensional lung tumor microenvironment modulates therapeutic compound responsiveness in vitro–implication for drug development. PLoS One.

[12] Durand RE (1990). Slow penetration of anthracyclines into spheroids and tumors: a therapeutic advantage?. Cancer Chemother Pharmacol.

[13] Kumar HR, Zhong X, Hoelz DJ, Rescorla FJ, Hickey RJ, Malkas LH (2008). Three-dimensional neuroblastoma cell culture: proteomic analysis between monolayer and multicellular tumor spheroids. Pediatr Surg Int.

[14] Wartenberg M, Fischer K, Hescheler J, Sauer H (2002). Modulation of intrinsic P-glycoprotein expression in multicellular prostate tumor spheroids by cell cycle inhibitors. Biochim Biophys Acta.

[15] Wartenberg M, Frey C, Diedershagen H, Ritgen J, Hescheler J, Sauer H (1998). Development of an intrinsic P-glycoprotein-mediated doxorubicin resistance in quiescent cell layers of large, multicellular prostate tumor spheroids. Int J Cancer.

[16] Minchinton AI, Tannock IF (2006). Drug penetration in solid tumours. Nat Rev Cancer.

[17] Hickman JA, Graeser R, de Hoogt R, Vidic S, Brito C, Gutekunst M (2014). IMI PREDECT Consortium: Three-dimensional models of cancer for pharmacology and cancer cell biology: capturing tumor complexity in vitro/ex vivo. Biotechnol J.

[18] Grantab R, Sivananthan S, Tannock IF (2006). The penetration of anticancer drugs through tumor tissue as a function of cellular adhesion and packing density of tumor cells. Cancer Res.

[19] Kwok TT, Twentyman PR (1985). The relationship between tumour geometry and the response of tumour cells to cytotoxic drugs–an in vitro study using EMT6 multicellular spheroids. Int J Cancer.

[20] Erlanson M, Daniel-Szolgay E, Carlsson J (1992). Relations between the penetration, binding and average concentration of cytostatic drugs in human tumour spheroids. Cancer Chemother Pharmacol.

[21] Carlsson J, Acker H (1988). Relations between pH, oxygen partial pressure and growth in cultured cell spheroids. Int J Cancer.

[22] Mueller-Klieser W, Freyer JP, Sutherland RM (1986). Influence of glucose and oxygen supply conditions on the oxygenation of multicellular spheroids. Br J Cancer.

[23] Freyer JP (1988). Role of necrosis in regulating the growth saturation of multicellular spheroids. Cancer Res.

[24] Olive PL, Banáth JP, Evans HH (1993). Cell killing and DNA damage by etoposide in Chinese hamster V79 monolayers and spheroids: influence of growth kinetics, growth environment and DNA packaging. Br J Cancer.

[25] Francia G, Man S, Teicher B, Grasso L, Kerbel RS (2004). Gene expression analysis of tumor spheroids reveals a role for suppressed DNA mismatch repair in multicellular resistance to alkylating agents. Mol Cell Biol.

[26] Borst P (1991). Genetic mechanisms of drug resistance. a review. Acta Oncol.

[27] Simons SM, Schindler M (1994). Cell biology mechanisms of multidrug resistance in tumors. Proc Natl Acad Sci U S A.

[28] Mattern J (2003). Drug Resistance in cancer: a multifactorial problem. Anticancer Res.

[29] Dangles V, Lazar V, Validire P, Richon S, Wertheimer M, Laville V (2002). Gene expression profiles of bladder cancers: evidence for a striking effect of in vitro cell models on gene patterns. Br J Cancer.

[30] Cavallaro U, Christofori G (2004). Cell adhesion and signalling by cadherins and Ig-CAMs in cancer. Nat Rev Cancer.

[31] Garrido C, Chauffert B, Pinard D, Tibaut F, Genne P, Assem M (1995). Circumvention of confluence-dependent resistance in a human multi-drug-resistant colon-cancer cell line. Int J Cancer.

[32] Wartenberg M, Ling FC, Müschen M, Klein F, Acker H, Gassmann M (2003). Regulation of the multidrug resistance transporter P-glycoprotein in multicellular tumor spheroids by hypoxia-inducible factor (HIF-1) and reactive oxygen species. FASEB J.

[33] Tomida A, Yun J, Tsuruo T (1996). Glucose-regulated stresses induce resistance to camptothecin in human cancer cells. Int J Cancer.

[34] Kolchinsky A, Roninson IB (1997). Drug resistance conferred by MDR1 expression in spheroids formed by glioblastoma cell lines. Anticancer Res.

[35] Ponce de León V, Barrera RR (2005). Changes in P-glycoprotein activity are mediated by the growth of a tumour cell line as multicellular spheroids. Cancer Cell Int.

[36] Olive PL, Durand RE, Banáth JP, Evans HH (1991). Etoposide sensitivity and topoisomerase II activity in Chinese hamster V79 monolayers and small spheroids. Int J Radiat Biol.

[37] de Lucio VM B, Barrera RR (2005). Characterization of human NSCLC cell line with innate etoposide-resistance mediated by cytoplasmic localization of topoisomerase II alpha. Cancer Sci.

[38] Oloumi A, MacPhail SH, Johnston PJ, Banáth JP, Olive PL (2000). Changes in subcellular distribution of topoisomerase IIalpha correlate with etoposide resistance in multicell spheroids and xenograft tumors. Cancer Res.

[39] Chikamori K, Grozav AG, Kozuki T, Grabowski D, Ganapathi R, Ganapathi MK (2010). DNA topoisomerase II enzymes as molecular targets for cancer chemotherapy. Curr Cancer Drug Targets.

[40] Drake FH, Hofmann GA, Bartus HF, Mattern MR, Crooke ST, Mirabelli CK (1989). Biochemical and pharmacological properties of p170 and p180 forms of topoisomerase II. Biochemistry.

[41] Cole SP (2014). Targeting multidrug resistance protein 1 (MRP1, ABCC1): past, present, and future. Annu Rev Pharmacol Toxicol.

[42] Cole SP, Deeley RG (2006). Transport of glutathione and glutathione conjugates by MRP1. Trends Pharmacol Sci.

[43] Borst P, Evers R, Kool M, Wijnholds J (2000). A family of drug transporters: the multidrug resistance-associated proteins. J Natl Cancer Inst.

[44] Takagi A, Watanabe M, Ishii Y, Morita J, Hirokawa Y, Matsuzaki T (2007). Three-dimensional cellular spheroid formation provides human prostate tumor cells with tissue-like features. Anticancer Res.

